# Decline of Phosphotransfer and Substrate Supply Metabolic Circuits Hinders ATP Cycling in Aging Myocardium

**DOI:** 10.1371/journal.pone.0136556

**Published:** 2015-09-17

**Authors:** Emirhan Nemutlu, Anu Gupta, Song Zhang, Maria Viqar, Ekhson Holmuhamedov, Andre Terzic, Arshad Jahangir, Petras Dzeja

**Affiliations:** 1 Division of Cardiovascular Diseases, Department of Internal Medicine, Mayo Clinic, Rochester, Minnesota, United States of America; 2 Center for Integrative Research on Cardiovascular Aging (CIRCA), Aurora University of Wisconsin Medical Group, Aurora Health Care, Milwaukee, Wisconsin, United States of America; Laurentian University, CANADA

## Abstract

Integration of mitochondria with cytosolic ATP-consuming/ATP-sensing and substrate supply processes is critical for muscle bioenergetics and electrical activity. Whether age-dependent muscle weakness and increased electrical instability depends on perturbations in cellular energetic circuits is unknown. To define energetic remodeling of aged atrial myocardium we tracked dynamics of ATP synthesis-utilization, substrate supply, and phosphotransfer circuits through adenylate kinase (AK), creatine kinase (CK), and glycolytic/glycogenolytic pathways using ^18^O stable isotope-based phosphometabolomic technology. Samples of intact atrial myocardium from adult and aged rats were subjected to ^18^O-labeling procedure at resting basal state, and analyzed using the ^18^O-assisted HPLC-GC/MS technique. Characteristics for aging atria were lower inorganic phosphate Pi[^18^O], γ-ATP[^18^O], β-ADP[^18^O], and creatine phosphate CrP[^18^O] ^18^O-labeling rates indicating diminished ATP utilization-synthesis and AK and CK phosphotransfer fluxes. Shift in dynamics of glycolytic phosphotransfer was reflected in the diminished G6P[^18^O] turnover with relatively constant glycogenolytic flux or G1P[^18^O] ^18^O-labeling. Labeling of G3P[^18^O], an indicator of G3P-shuttle activity and substrate supply to mitochondria, was depressed in aged myocardium. Aged atrial myocardium displayed reduced incorporation of ^18^O into second (^18^O_2_), third (^18^O_3_), and fourth (^18^O_4_) positions of Pi[^18^O] and a lower Pi[^18^O]/γ-ATP[^18^ O]-labeling ratio, indicating delayed energetic communication and ATP cycling between mitochondria and cellular ATPases. Adrenergic stress alleviated diminished CK flux, AK catalyzed β-ATP turnover and energetic communication in aging atria. Thus, ^18^O-assisted phosphometabolomics uncovered simultaneous phosphotransfer through AK, CK, and glycolytic pathways and G3P substrate shuttle deficits hindering energetic communication and ATP cycling, which may underlie energetic vulnerability of aging atrial myocardium.

## Introduction

Vigorous atrial function is critical for sustaining normal heart work and uninterrupted blood flow yet it declines with aging increasing susceptibility to atrial fibrillation (AF) [1,2]. Whether a change in dynamics of atrial energetics contributes to functional decline and its significance in aging process is unknown [1–5]. In ventricles, coordination of contractile and electrical activities of myocardium depends on the integrated energetic signaling system that ensures optimal substrate supply, generation of ATP, and delivery of high-energy phosphoryls to cellular ATPases subsequently conveying energy demand signals to mitochondrial ATP production [6–12]. In recent years, new evidence has accumulated that phosphotransfer circuits composed from creatine kinase (CK), adenylate kinase (AK), and glycolytic/glycogenolytic enzymes along with substrate shuttles, such as glycerol-3-phosphate (G3P), are essential parts of the cardiac bioenergetic infrastructure integral to maintaining energy homeostasis [11,13–17]. The failing ventricle myocardium is characterized by reduction of high-energy phosphates and lower activity of the phosphotransfer enzymes CK and AK which facilitate transfer of high-energy phosphoryls and their metabolites from sites of production to sites of utilization [7,9,10,18–20]. These phosphotransfer systems serve also as metabolic signal transducers, coupling the cell energetic status to ion channel function and membrane excitability [21–24]. Disruption of energetic signaling pathways or ion channels with metabolic-sensing properties predisposes the myocardium to electrical instability [21,22,25–27]. In humans with chronic AF discrete defects in cellular and mitochondrial energetics develop in the atrial myocardium, suggesting a potential link between metabolic derangements and electrical perturbations [28–33]. Enzyme activities of CK and AK tightly correlated with ATP concentration and AF duration, implying that impairment in atrial bioenergetics may contribute to the substrate for AF [7,18,34]. However, the significance of changes in metabolic flux through atrial phosphotransfer systems in aging myocardium has not been determined.

Mitochondria function and regulation of mitochondrial biogenesis decline with the aging process, which results in increased reactive oxygen species and decreased ATP synthesis [35–38]. Moreover, changes in dynamics of ATP delivery and hindered energetic communication between ATPases and mitochondria and metabolic signaling to metabolic sensors can worsen situation yet the significance of altered energetic dynamics has not been determined. This is of importance since metabolic perturbations can trigger energy-driven oscillations in potassium currents producing cyclical changes in the cardiac action potential that may underlie to the genesis of arrhythmias, fibrillation and cardiac arrest [27,39–42].

The aim of this study was to gain insights into the fine mechanisms of altered dynamics of energy metabolism in aging myocardium by revealing age-dependent perturbations in the atrial bioenergetics system coupled with phosphotransfer pathway and ATP cycling rearrangements. Age-dependent shift of cellular energetics and phosphotransfer dynamics in rat atria was determined using ^18^O-labeling phosphometabolomic methodology and mass spectrometry specifically design to study small myocardial samples which can be applied to human myocardium [15,17,43,44]. Our study uncovered age-dependent decline in phosphometabolite turnover rates and dynamic energetic rearrangements in aging rat atria with simultaneous phosphotransfer circuit and mitochondrial substrate shuttle deficits hindering ATP cycling that may underlie the vulnerability of the whole energetic system, muscle weakness and electrical instability.

## Materials and Methods

### Animal Models

Fischer 344 rats, 6 months (adult) and 24 months (aged), were used in this study. Fischer 344 rats (obtained from the National Institute of Aging) were maintained on a standard chow diet and housed in a controlled environment for at least 1 week before being sacrificed. Rats were anesthetized with intraperitoneal injection of sodium pentobarbital (50 mg/kg). All experimental procedures were designed in accordance with the National Institutes of Health guidelines and were approved by the Mayo Institutional Animal Care and Use Committee.

### ^18^O Isotopic Labeling of Cellular Phosphoryls

Atrial tissues were removed surgically from excised hearts and washed and preincubated in oxygenated Krebs-Henseleit (KH) solution. Subsequently, samples of intact atrial myocardium from adult and aged rats were subjected to metabolite ^18^O-labeling procedure [17,44,45]. Briefly, atrial tissue samples were transferred to oxygenated KH solution enriched with 30% of H_2_O[^18^O] (Isotec Inc) and incubated for 1 minute then quickly freeze-clamped into liquid nitrogen and stored for ^18^O-labeling and biochemical analyses. Separately, atrial tissues were incubated in KH solution enriched with ^18^O and isoproterenol (ISO) (0.001 μM) for 1 minute for ^18^O-labeling to simulate oxidative and metabolic stress conditions [46,47]. The tissue was frozen immediately in liquid nitrogen and stored at –80°C until further analysis of ^18^O-labeling by mass spectrometry. This protocol can be used on small human atrial myocardium samples, which studies are underway.

### Purification and Isotopic Analysis of ^18^O-Labeled Cellular Phosphoryls

**Fig 1:**
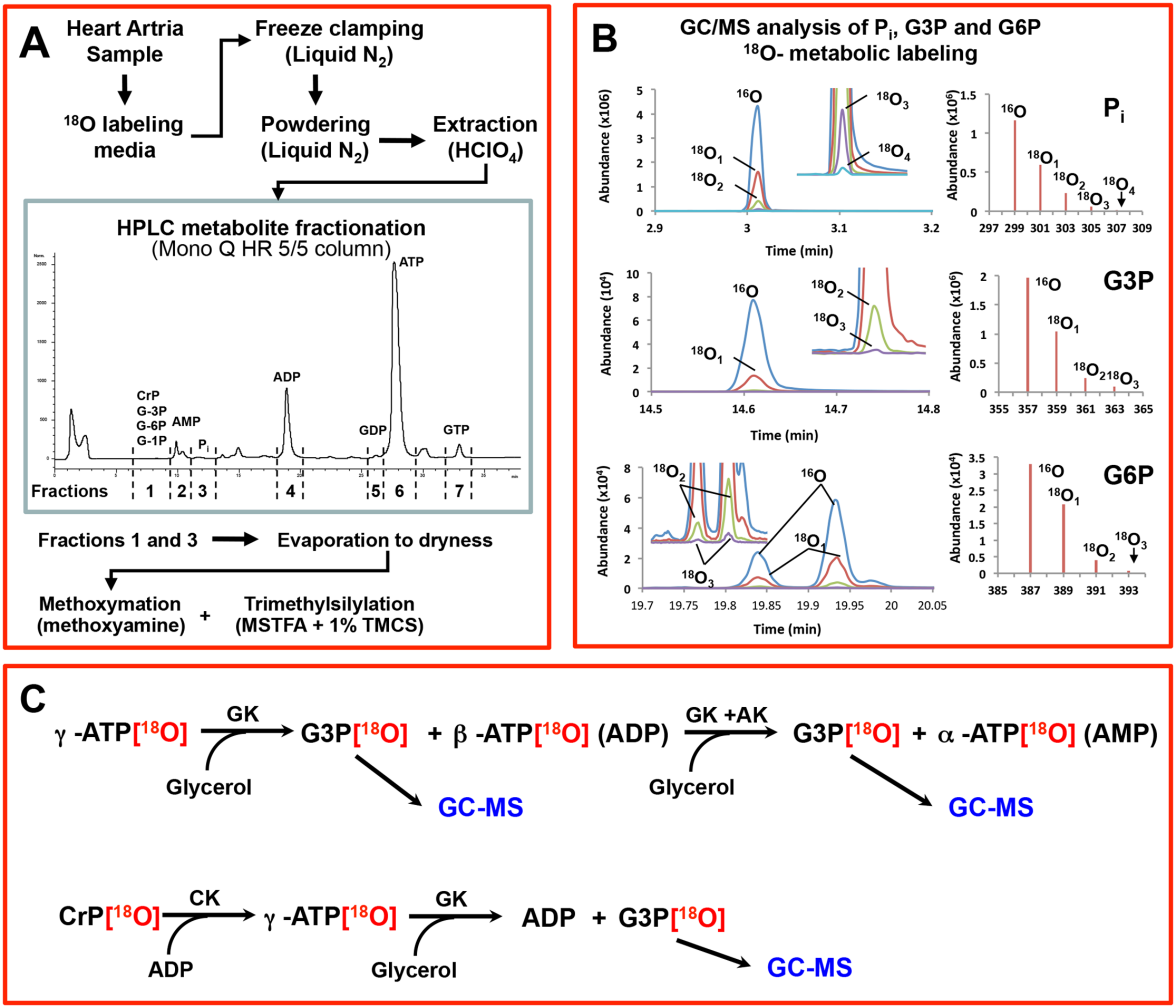
^18^O-labeling analysis of phosphometabolites using HPLC and ^18^O-assisted GC/MS. A, Sample preparation and fractionation for GC/MS analysis. B, Analysis of Pi, G3P, and G6P using GC/MS. C, Enzymatic reactions for γ- and β-ATP and CrP to analyze their ^18^O-metabolic–labeling ratio with GC/MS as G3P. AK indicates adenylate kinase; CrP, creatine phosphate; G3P, glycerol-3-phosphate; G6P, glucose-6-phosphate; Pi, inorganic phosphate.

Atrial samples were freeze-clamped and pulverized in mortar with liquid nitrogen, and extracted in a solution containing 0.6 M HClO_4_ and 1 mM EDTA. The samples were centrifuged at 10,500 rpm for 10 minutes at 4°C to precipitate proteins. The pellet then was left in 2N NaOH for protein estimation and the supernatants were neutralized using 2M KHCO_3_ until neutral (pH>7.0). The supernatants were left on ice for 30 minutes in a cold room. Then samples were centrifuged at 3000 rpm for 10 minutes to precipitate KClO_4_. The supernatants were stored at –20°C until they were analyzed using HPLC for fractionation and subsequent ^18^O-assisted GC/MS for ^18^O-enrichment analyses (Fig 1A and 1B). Metabolites were analyzed with a Hewlett-Packard 5980B/5973 gas chromatograph mass spectrometer and data was analyzed using Chemstation software. Cellular phosphometabolites were purified and quantified with HPLC HP 1100 (Fig 1A) using a Mono Q HR 5/5 ion-exchange column (Pharmacia Biotech) with triethylammonium bicarbonate buffer (pH 8.8 at 1 mL/min flow rate) [10,19,48,49]. From each sample, 4 fractions were collected. The first fraction contained glucose-6-phosphate (G6P), glycerol-3-phosphate (G3P), and glucose-1-phosphate (G1P), and the second through fourth fractions contained inorganic phosphate (P_i_), ADP, and ATP, respectively (Fig 1A). Fractions were dried out using vacuum centrifugation (SpeedVac, Savant) and reconstituted with water. Pi, G3P, G1P, and G6P reconstitutions were transferred to GC/MS vials for silylation (Fig 1B), while the β-phosphoryl of ADP, γ- and β-phosphoryl of ATP and phosphoryl of creatine phosphate (CrP) were analyzed after enzymatic transfer of corresponding phosphoryls to glycerol (Fig 1C) [19,49,50]. Samples that contained phosphoryls of γ-ATP, β-ATP, β-ADP as G3P, Pi, G6P, G1P, and G3P were converted to respective trimethylsilyl derivatives with Tri-Sil/BSA (Pierce) as the derivatization agent [10,49,51]. The ^18^O enrichments of phosphoryls were determined with GC/MS operated in the select ion-monitoring mode. GC/MS analysis of Pi, G3P, and G6P ^18^O-labeling is presented in Fig 1B. Left panel represents GC/MS chromatograms of metabolites, while in the right panel oxygen the isotope abundance is shown. Using this approach in a single run the metabolic dynamics of glycolysis (G6P) and glycogenolysis (G1P) and mitochondrial substrate shuttle activity (G3P) can be monitored.

The cumulative percentage of phosphoryl oxygens replaced by ^18^O in the metabolites was calculated using the formula [17,51]:

$$\left[{\%}^{18}{\text{O}}_{1}+2({\%}^{18}{\text{O}}_{2})\;+\;3({\%}^{18}{\text{O}}_{3})+\;\ldots .\text{ n(}\%{\text{m}}^{18}{\text{O}}_{\text{n}}\text{)}]/[\text{n(}\%{\;}^{18}\text{O in }{\text{H}}_{2}\text{O)}\right]$$

where n is the total number of phosphoryl oxygen sites in the metabolite. Calculation of turnover rates has been described in detail previously [17,49,51–53]. Briefly, phosphometabolites turnover times were calculated using the formula:

$$p_{t}\left(\text{phosphometabolite}\right)=\left({1-2}^{-N}\right)´\text{p}\left(\left[{}^{18}\text{O}\right]{\text{H}}_{2}\text{O}\right)$$

where *p*_*t*_(phosphometabolite) is a fraction of ^18^O-labeled phosphometabolite at given time *t*, N is equal to the number of turnover cycles observed during incubation period, and p([^18^O]H_2_O) is a fraction of ^18^O in media water [49,52–54].

### Statistical Analysis

Data are expressed as mean±SE. The Student *t* test for unpaired samples was used for statistical analysis and a difference at *P*<0.05 was considered significant.

## Results

### Aging-Associated Changes in Atrial ATP Synthesis, Consumption, and Phosphotransfer Dynamics

**Fig 2:**
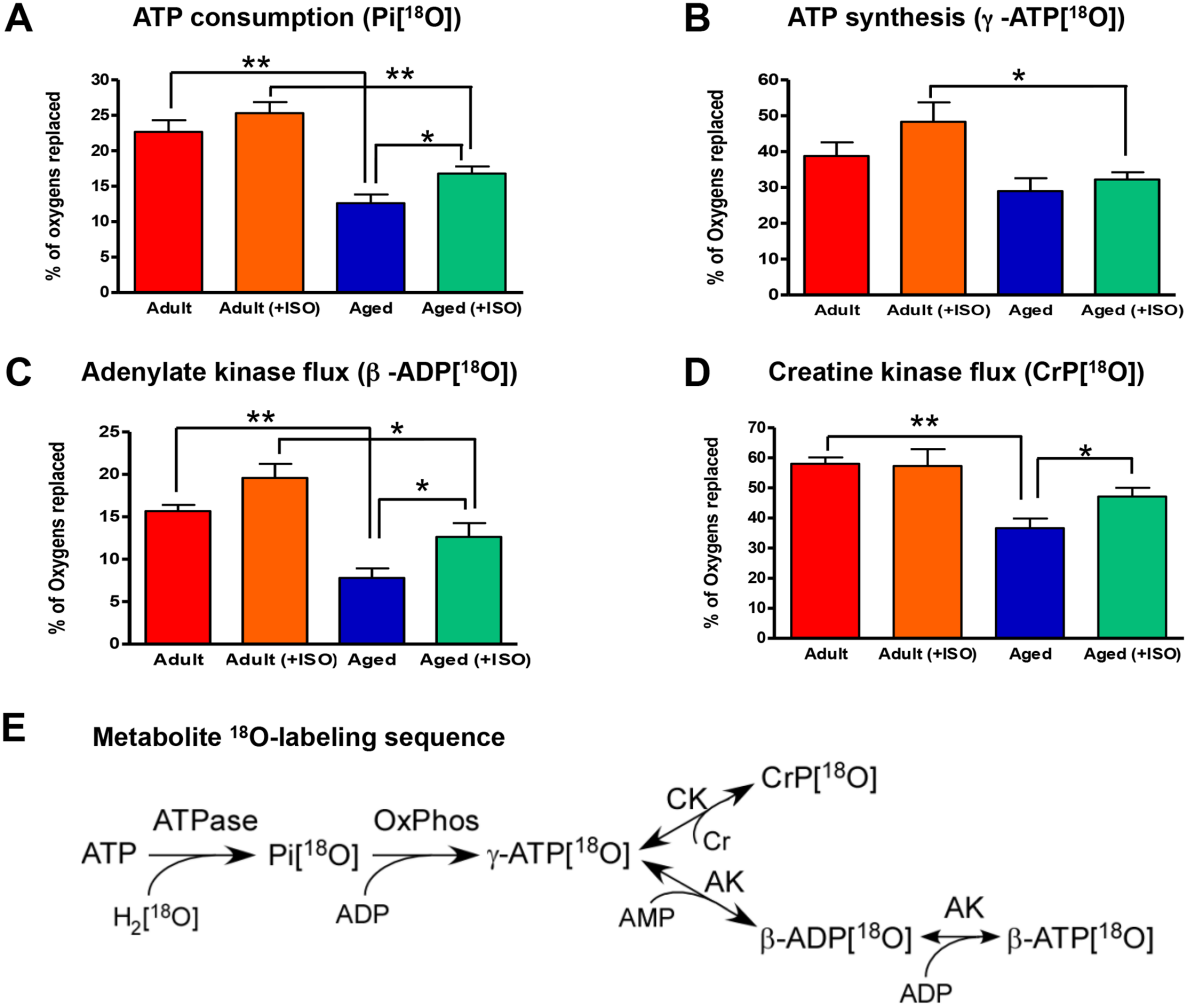
Aging-associated changes in atrial Pi, γ-ATP, β-ADP, and CrP ^18^O-metabolic–labeling reflecting altered ATP consumption and synthesis processes, and AK and CK velocities. A, Aging and stress (ISO) effects on atrial Pi turnover, indicators of ATP consumption rate. B, Aging and stress effects on atrial ATP γ-phosphoryl turnover, indicators of ATP synthesis rate. C, Aging and stress effects on atrial ADP β-phosphoryl turnover, indicators of AK metabolic flux. D, Aging and stress effects on atrial CrP turnover, indicators of CK metabolic flux. E, Schematic representation of ^18^O-labeling reaction sequence. *P<0.05 and **P<0.01. AK indicates adenylate kinase; CK, creatine kinase; CrP, creatine phosphate; ISO, isoproterenol; Pi, inorganic phosphate.

Vigorous ATP consumption and synthesis cycle is critical in maintaining cellular energy homeostasis. Knowledge of basal metabolic state, which is independent of contractile activity, is a valuable parameter for understanding remodeling of energy metabolism during aging. Here, we determined metabolite turnover rates in basal state which is more stable and not confounded by variation in contractile activity. With aging, ATP consumption rate of intact atrial myocardium (Fig 2A), as assessed by ^18^O incorporation into Pi during ATP hydrolysis, was significantly depressed. Specifically, Pi ^18^O-labeling rate decreased from 22.7±1.6 in adult to 12.6±1.2%^18^O/min (*P<*0.01, n = 6) in the aged atrial myocardium. Presence of ISO and metabolic stress did not produce a significant effect on ATP consumption and Pi ^18^O-labeling rate in the adult atrial tissue. However, ISO had a significant effect on restoring depressed ATP consumption in aged atria (Fig 2A and S1 Table). Specifically, Pi ^18^O-labeling rate of aged myocardium increased to 16.8±1.0%^18^O/min, or by 33%, in the presence of ISO compared to the aged atria without ISO (*P*<0.05, n = 6). Despite significant improvement in ATP consumption, the difference in Pi ^18^O-labeling rate between adult (+ISO) (25.3±1.6%^18^O/min) and aged (+ISO) (16.8±1.0%^18^O/min) still were significant at *P*<0.01.

ATP synthesis rate, as assessed by the rate of γ-ATP ^18^O-labeling which takes place mostly in mitochondria, was lower in aged atrial myocardium at 29.0±3.3%^18^O/min compared to 38.8±3.8%^18^O/min in adult; however, it do not reach statistical significance (Fig 2B and S1 Table). Significant reduction in ATP synthesis rate between adult and aged atria was observed only in the presence of ISO. The γ-ATP ^18^O-labeling rate was decreased from 48.3±5.4 in adult (+ISO) to 32.2±1.8%^18^O/min in aged (+ISO) atria (*P*<0.05, n = 6).

AK metabolic flux, as assessed by β-ADP ^18^O-labeling, was significantly lower in aged atrial myocardium (Fig 2C and S1 Table). The rate of β-ADP ^18^O-labeling was decreased from 15.7±0.7%^18^O/min in adult to 7.8±1.1%^18^O/min in aged rat atria (*P*<0.01, n = 6). The presence of ISO significantly improved AK phosphotransfer in aged atrial myocardium to 12.6±1.6%^18^O/min, which was significant compared to the absence of ISO (*P*<0.05). Despite enhancement by ISO, AK flux was still significantly depressed in aged (+ISO) compared to adult (+ISO) atria (*P*<0.05, n = 6).

CK metabolic flux, as assessed by CrP ^18^O-labeling, was significantly lower in aged atrial myocardium (Fig 2D and S1 Table). The rate of CrP ^18^O-labeling was decreased from 60.2±0.6%^18^O/min in adult to 39.1±2.7%^18^O/min in aged rat atria (*P*<0.01, n = 4–8). The presence of ISO significantly improved CK phosphotransfer in aged atrial myocardium to 48.4±3.0%^18^O/min, which was significant compared to without ISO (*P*<0.05, n = 6–8). Due to improvement by ISO, CK flux was no longer statistically significantly depressed in aged (+ISO) compared to adult (+ISO) atria.

### Aging-Dependent Changes in Atrial Glycolytic, Glycogenolytic, and Substrate Shuttle Activities

**Fig 3:**
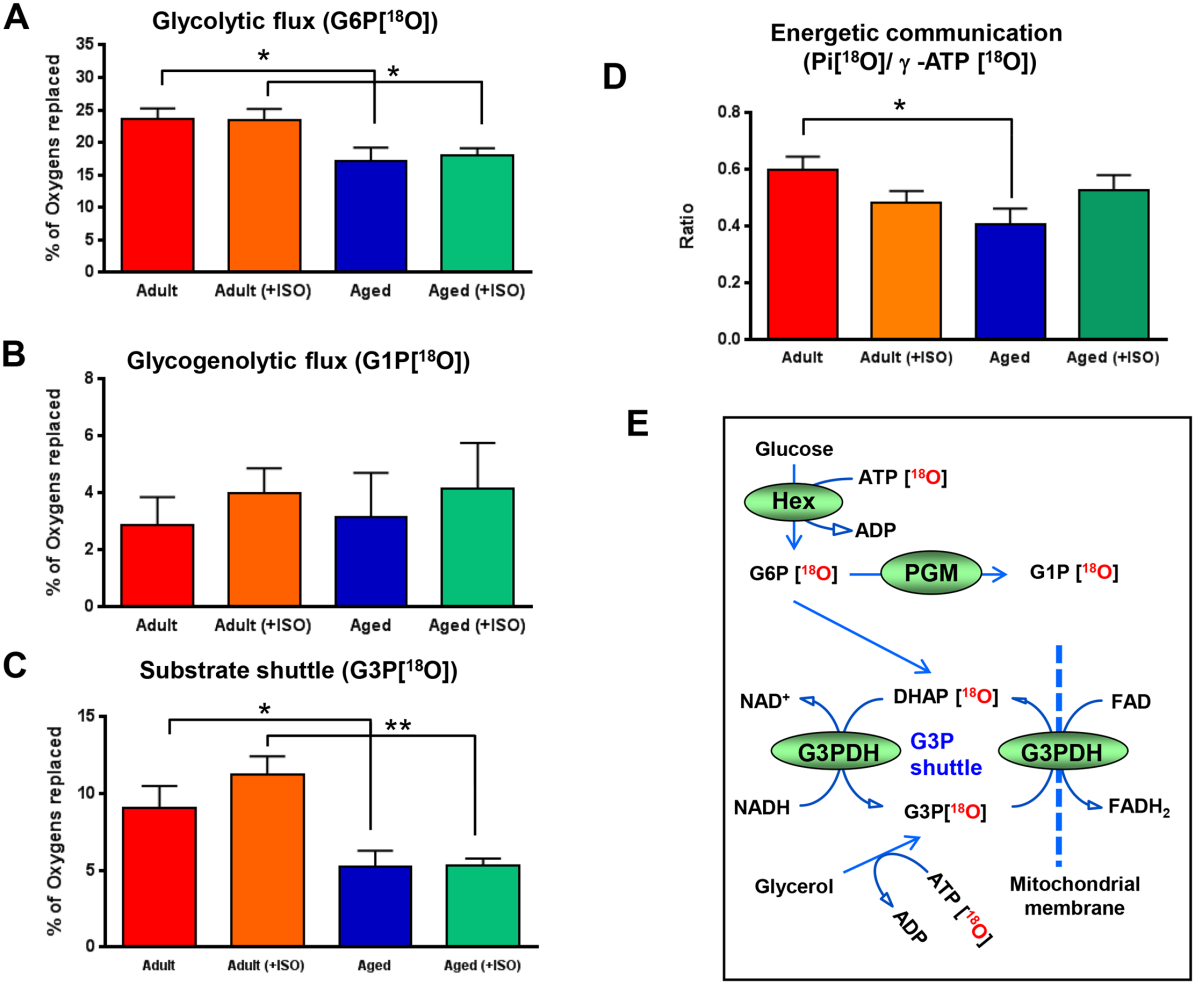
Aging-associated changes in atrial G6P, G1P, and G3P ^18^O-metabolic–labeling indicating alterations in glycolytic, glycogenolytic, and substrate shuttle activities. A, Aging and stress (ISO) effects on atrial G6P turnover, indicators of glycolytic rate. B, Aging and stress effects on atrial G1P turnover, indicators of glycogenolytic rate. C, Aging and stress effects on atrial G3P turnover, indicators of substrate shuttle activity. D, Aging and stress (ISO) effects on atrial Pi/γ-ATP ^18^O-labeling ratio, indicators of energetic communication between ATP consumption and ATP production processes. E, Schematic representation of reaction sequences and metabolite^18^O-labeling allowing to track glycolytic, glycogenolytic, and α-glycerophosphate substrate shuttle dynamics. * *P*<0.05 and ** *P*<0.01. G1P indicates glucose-1-phosphate; G3P, glycerol-3-phosphate; G6P, glucose-6-phosphate; ISO, isoproterenol; Pi, inorganic phosphate.

Intracellular spatially arranged glycolytic and glycogenolytic networks, in addition to their energy (ATP)-producing role, have the robust capability to catalyze high energy phosphoryl exchange and distribution from cellular sites of ATP generation in mitochondria to ATP consumption providing energy to remote cellular processes [17,51,55]. Here, glycolytic and glycogenolytic net phosphotransfer fluxes were monitored by measuring the rate of appearance of ^18^O-labeled phosphoryl species in G6P[^18^O] and G1P[^18^O], respectively (Fig 3A, 3B and S1 Table). G6P ^18^O-labeling and glycolytic flux was significantly reduced from 23.6±1.6%^18^O/min to 17.2±1.9%^18^O/min, or by 27% (*P<*0.05, n = 5–6), in aged rat atria, while G1P ^18^O-labeling, reflecting glycogenolytic phosphotransfer flux, which was 2.87±0.9%^18^O/min and 3.18±1.4%^18^O/min in adult and aged rat atria (n = 5–6), respectively, was not affected by aging. Metabolic stress induced by ISO had no major effect on both glycolytic and glycogenolytic phosphotransfer fluxes except a trend of higher glycogenolytic flux in the presence of ISO in both adult and aged atria, which was equal to 4.0±0.8 and 4.15±1.5%^18^O/min (n = 5–6), respectively.

Alpha-glycerophosphate substrate shuttle plays a pivotal role in cellular bioenergetics by linking cytosolic metabolic networks to mitochondrial oxidations [56]. Here, the G3P shuttle activity was examined by measuring the rate of appearance of ^18^O-labeled phosphoryl species in G3P[^18^O] (Fig 3C and S1 Table). Labeling of G3P by ^18^O was reduced by 42% from 11.01±1.70 in adult to 6.62±1.08%^18^O/min in the aged rat atria (*P<*0.05, n = 6–8). The difference between adult and aged atria was more significant in the presence of metabolic stress induced by ISO. Under these conditions, G3P ^18^O-labeling and shuttle activity was reduced by 53% from 11.44±0.84 in adult to 5.68±0.50%^18^O/min in aged rat atria (*P<*0.01, n = 6–8).

### Aging-Associated Depression of Atrial ATP Cycling and Energetic Communication

Kinetics of ^18^O-labeling of Pi at ATPase site and γ-ATP at mitochondrial site and resulting Pi[^18^O]/γ-ATP[^18^O] ratio is an indicator of energetic communication between intracellular ATP consumption and ATP production processes.[17,44] The Pi/γ-ATP ^18^O-labeling percentage ratio was significantly reduced from 0.60±0.05 in adult to 0.41±0.05 (*P<*0.05, n = 4–6) in aged rat atria, indicating impediment of energetic communication between mitochondria and cellular ATPases in aging myocardium (Fig 3D and S1 Table). In the presence of ISO and metabolic stress, the Pi[^18^O]/γ-ATP[^18^O] ratio was improved in the aged atria, and there was no significant difference compared to adult atria. Thus, ^18^O-labeling technology permits tracking intracellular energetic communication along with glycolytic, glycogenolytic, and substrate shuttle dynamics (Fig 3E and S1 Table).

### Aging-Associated Changes in ATP Turnover Cycles and Metabolic Pathways

**Fig 4:**
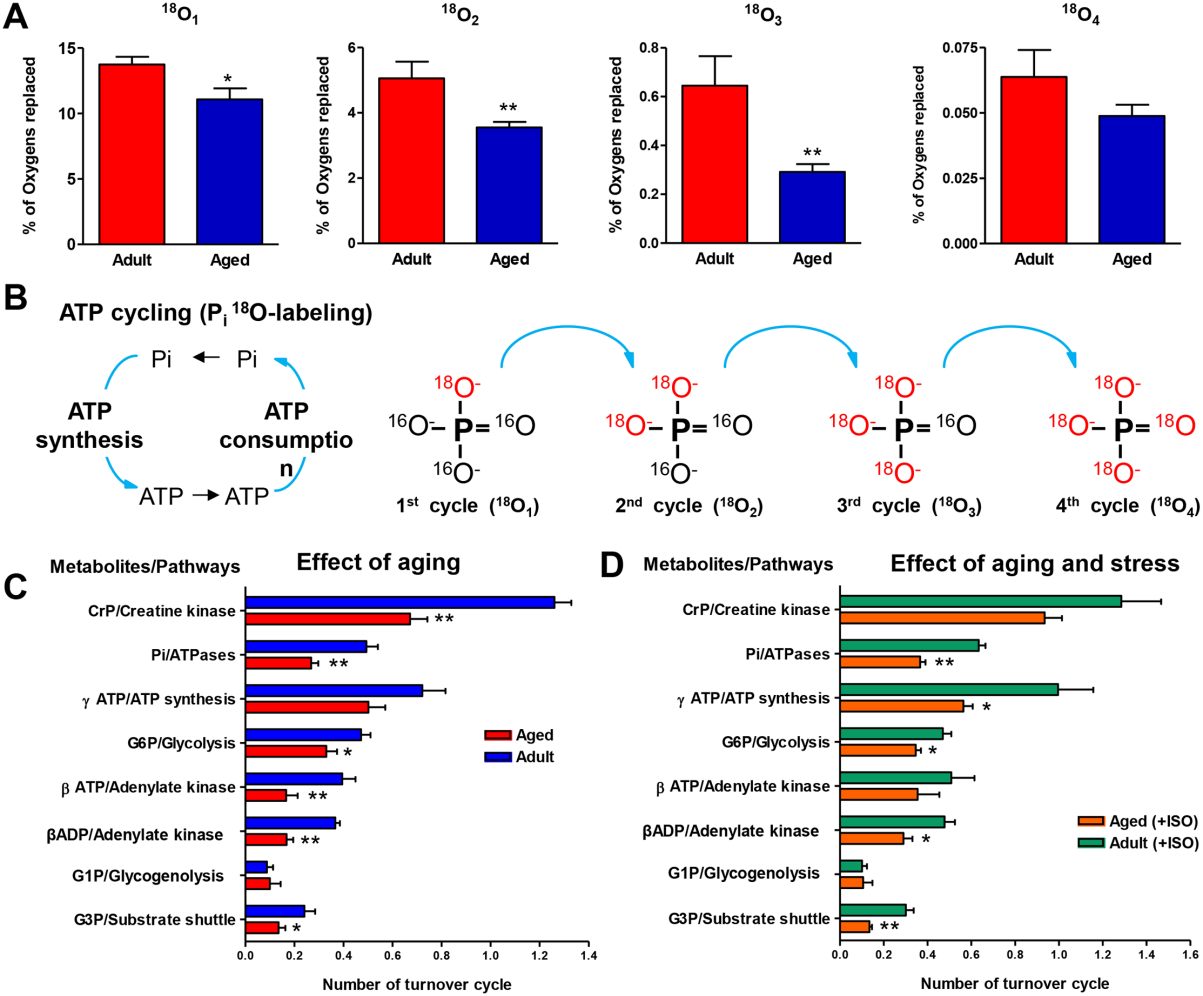
Aging-associated decline in atrial ATP and Pi cycling between ATP synthesis and ATP consumption sites and stress effect on energetic pathways. A, Aging effects on first, second, third, and fourth ^18^O-atom incorporation into Pi signifying cycles of ATP/Pi exchange between consumption and synthesis sites. B, Schematic representation of ^18^O-labeling during ATP cycling. C, Aging effects on atrial metabolite turnover rates and corresponding pathways. D, Aging and stress effects on atrial energy metabolite turnover rates and corresponding pathways. * *P*<0.05 and ** *P*<0.01. CrP indicates creatine phosphate; G1P, glucose-1-phosphate; G3P, glycerol-3-phosphate; G6P, glucose-6-phosphate; ISO, isoproterenol; Pi, inorganic phosphate.

Diminished ATP turnover in aging myocardium could be a result of reduced ATPases and ATP synthases as well as hindered transfer and cycling of ATP between mitochondria and sites of ATP utilization [9,16]. Stable isotope ^18^O–labeling technology uniquely permits tracking of ATP and Pi cycling between cellular ATPases and sites of ATP regeneration in mitochondria in intact tissue [44]. ATP cycling and energetic communication can be monitored by the incorporation of ^18^O into first (^18^O_1_), second (^18^O_2_), third (^18^O_3_) and fourth (^18^O_4_) positions of Pi (Fig 4A and S2 Table). The rate of incorporation of ^18^O into different positions of Pi indicates how fast ^18^O-labeled Pi species produced during ATP hydrolysis can reach mitochondria and get back to ATPases as γ-ATP[^18^O], to get second, third, and fourth ^18^O atoms incorporated during cycles of ATP hydrolysis (Fig 4B). The results demonstrate that aging atrial myocardium has a lower rate of incorporation of ^18^O into second (^18^O_2_), third (^18^O_3_), and fourth (^18^O_4_) positions of Pi (Fig 4A). Specifically, the percentage of oxygens replaced in ^18^O_1_ position was reduced from 13.7±0.58 in control (n = 10) to 11.1±085 in aging atria (*P*<0.05, n = 17); in ^18^O_2_, from 5.1±0.51 to 3.6±0.17 (*P<*0.01); in ^18^O_3_, from 0.64±0.12 to 0.29±0.03 (*P*<0.01); and in ^18^O_4_, from 0.06±0.01 to 0.05±0.00 (*P>*0.05). Thus, cycling of Pi and ATP between cellular ATPases and mitochondria is compromised in aged atria myocardium.

Metabolite turnover and pathway data analysis (Fig 4C and S3 Table) further revealed dynamic rearrangements in the aging atrial myocardial energetic system with diminished CK, AK, and glycolytic phosphotransfer rates, and ATPase velocity and mitochondrial substrate shuttle function. Specifically, CrP turnover, reflecting CK phosphotransfer rate, was reduced from 1.26±0.06 in control to 0.67±0.08 in aging atria (*P*<0.01, n = 4–8). β-ATP and β-ADP turnovers, indicating AK phosphotransfer velocity, were reduced from 0.40±0.05 and 0.37±0.02 in control to 0.17±0.03 and 0.17±0.04 in aging atria, respectively (*P*<0.01, n = 5–6). Pi turnover, reflecting ATPase rate, was reduced from 0.49±0.05 in control to 0.27±0.03 in aging atria (*P*<0.01, n = 5–6). G6P and G3P turnovers, indicating glycolytic phosphotransfer and substrate shuttle activity, respectively, were reduced from 0.47±0.04 and 0.24±0.04 in control to 0.33±0.04 and 0.14±0.03 in aging atria (*P*<0.05, n = 5–6). G1P turnover, an indicator of glycogenolysis, was not different between control and aging myocardium.

Metabolite turnover and pathway analysis in the presence of metabolic stress (+ISO) (Fig 4D and S3 Table) revealed a positive effect of ISO on CrP and β-ATP/ADP turnovers, reflecting CK and AK velocities, respectively, in aging myocardium compared to without ISO and control adults. In the presence of ISO, CrP turnover was increased to 0.93±0.09 (n = 6) in aging atria with no effect on adult myocardium (not shown). β-ATP and β-ADP turnovers were almost doubled by increasing to 0.36±0.09 and 0.29±0.04 (n = 6) in the presence of ISO. In this regard, aging myocardium had higher Pi and ATP turnovers in the presence of ISO and trend to increase in γ-ATP turnover or ATP synthesis rate, indicating potential of stress response and adaptability of the energetic system.

## Discussion

Aging imposes structural and metabolic alterations in atrial myocardium and increases risk to AF [1,2,7,29]. Defining metabolic mechanisms of aging is necessary for designing interventions to improve human health span, quality of life and prevention of associated diseases [57]. Here, age-dependent shift of cellular energetics and phosphotransfer kinetics of atrial myocardial samples were determined using advanced ^18^O-labeling phosphometabolomic methodology and mass spectrometry [15,44]. To get a broader picture of rearrangements in the energetic system and insights into mechanisms, turnover rates of Pi[^18^O] (an indicator of ATP utilization), γ-ATP[^18^O] (an indicator of ATP synthesis), β-ATP[^18^O] and β-ADP[^18^O] (indicators of AK flux), CrP[^18^O] (an indicator of CK flux), G6P[^18^O] (an indicator of glycolytic flux), G1P[^18^O] (an indicator of glycogenolytic flux), and G3P[^18^O] (an indicator of substrate shuttle activity) were determined using ^18^O-assisted mass spectrometry[15,17,44,58].

Using stable isotope ^18^O–assisted dynamic metabolic profiling, we have uncovered developing simultaneous ATP cycling, phosphotransfer, and mitochondrial substrate shuttle deficits in aging myocardium. Aged atrial myocardium had significant lower ATP turnover rate which was significantly potentiated by applying adrenergic stress. This indicates that reduced β-adrenergic signaling and Ca^2+^ cycling may preclude activation of mitochondrial enzymes in aging atrial myocardium limiting ATP turnover. Significant reduction in ATP synthesis rate between adult and aged atria was still evident in the presence of ISO, indicating confounding defects in protein levels and gene expression [35,36,59]. The observation that stress has a significant effect on the aged myocardium could also be a link to higher susceptibility to attacks of aged hearts which have lower energetic capacity [37,38,60].

In the aging atria, both CK and AK phosphotransfers, which are responsible for distributing high-energy phosphoryls, were significantly depressed. The presence of adrenergic stress improved both CK and AK phosphotransfers in aged myocardium, indicating regulatory potential of β-adrenergic signaling. Due to improvement, CK flux was no longer statistically significantly depressed in aged atria. Specific molecular mechanisms of such improvement remain to be determined, although they can include changes in posttranslational modification of enzymes. Previous our study did not reveal significant transcriptomic and proteomic changes in AK and CK levels [38]. Changes in AK and CK flux could be related to posttranslational modification, as there is an increase in AK1 is carbonylation and CK nitration with aging [61,62]. Beside high-energy phosphoryl transfer, the high CK- and AK-mediated catalysis is necessary to maintain intact myocardial phosphoryl-carrying molecule pools, apparently through rapid rephosphorylation of them, thus preventing loss of molecules through the degradation and/or efflux pathways [9,15,18,63]. In heart failure, depressed phosphotransfer enzyme activities correlate with reduced tissue ATP levels, whereas CrP levels inversely relate with atrial and ventricular load [18].

The turnover of G3P, which connects glycolytic and mitochondrial metabolism, was also significantly depressed in aged atrial myocardium, indicating deficient activity of G3P shuttle and substrate supply to mitochondria [44,56]. Of note, the difference in G3P turnover between adult and aged atria was more significant in the presence of metabolic stress. In this regard, critical significance of G3P turnover is indicated by mutations in G3P dehydrogenase 1-like (GPD1-L) protein, which is highly expressed in the heart, that are linked to Brugada and sudden infant death syndromes characterized by vulnerability to metabolic stress [64–66]. G3P shuttle is important and underappreciated component in cellular energetic system. We demonstrate for the first time that it is depressed in aging. Concomitantly, G6P turnover was depressed in aged atrial myocardium, too, indicating deficient glycolytic phosphotransfer and hexokinase catalyzed shuttling of ATP from mitochondria to cellular ATPases. G1P turnover, an indicator of phosphoryl transfer in glycogenolysis, was not changed in aging atria compared to control. Applied metabolic stress had no major effect on both glycolytic and glycogenolytic phosphotransfer fluxes. Thus, ^18^O stable isotope–resolved metabolite dynamics provide a systemic view of deficits and rearrangements in the energetic system of aging atrial myocardium, uncovering most vulnerable steps.

Cardiac contractile function depends not only on the rate of delivery of high-energy phosphoryls (ATP, CrP) but also on removal of the end products of the ATPase reaction (ADP, Pi, and H^+^) and conveying metabolic signals to ATP generation sites [9,17]. Specifically, ^18^O-labeled Pi species produced during ATP hydrolysis at an ATPase site must reach a distinct ATP production site to be incorporated into γ-ATP. Delay in activation of ATP production will result in different kinetics of γ-ATP ^18^O-labeling compared to that of Pi[^18^O]. Thus, by following Pi and γ-ATP ^18^O-labeling kinetics, intracellular energetic communication can be monitored. Indeed, the Pi/γ-ATP ^18^O-labeling ratio was significantly reduced in aged rat atria, indicating impediment of energetic communication between mitochondria and cellular ATPases [17,67]. Metabolic stress and associated increase in Ca^2+^ mobilizes cellular energetic resources and activates number of enzymatic process [68]. In the presence of metabolic stress, the Pi[^18^O]/γ-ATP[^18^O] ratio and energetic communication was significantly improved in aged atria compared to adult. This was associated with alleviation of some key energetic parameters after adrenergic stress in aging myocardium including creatine kinase flux, adenylate kinase catalyzed β-ATP turnover, Pi/ATP turnover and energetic communication. Taken together, these results indicate potential of improvement of aging myocardial bioenergetics by metabolic stress training.

Optimal functioning and the rate of communication between components of the cellular bioenergetic system are supported by complementation in phosphotransfer enzyme activity and intimate interaction of phosphotransfer proteins with cellular sites of ATP utilization, metabolic sensing (K-ATP, AMPK) and energy transduction (mitochondria, glycolysis) [6,11,16,23,24]. Using advantages of ^18^O-labeling technology, we demonstrate that aging atrial myocardium has lower rates of incorporation of ^18^O into separate positions of Pi, indicating diminished cycling of Pi and ATP between cellular ATPases and mitochondria. This could be due to rearrangements in the aging atrial energetic system with diminished CK, AK, and glycolytic phosphotransfer rates, and ATPase velocity and mitochondrial substrate shuttle function. Previous our gene array data show reduced transcript levels of genes in ATP and G3P metabolism in aged hearts [38]. In addition, transcript levels of mitochondrial Complex I nDNA encoded genes in the aged hearts was associated with functional decline and a 46% reduction in enzymatic activity as determined by the rotenone-sensitive reduction of ubiquinone-1 and decreased state 3 respiration in malate-pyruvate NAD-dependent substrate [38]. No significant changes in Complex II activity in the aged hearts were found as determined by the reduction of ubiquinone-2 by succinate.

The data presented here is along with the concept of cardiac bioenergetic infrastructure, consisting of coupled mitochondrial, glycolytic, and phosphotransfer networks, which are arranged to maintain energy homeostasis by ensuring tight energy supply-demand match, force-frequency, and mechano-electrical coupling relationships [6,11,15,16,59,69]. According to this concept, the AK, CK, and glycolytic/glycogenolytic phosphotransfer circuits along with glycerophosphate shuttle are essential parts of myocardial bioenergetics infrastructure. These enzymatic conduits provide energetic continuum by distributing high-energy phosphoryls to cellular ATPases, maintaining high ΔG for ATP hydrolysis and conveying energy demand signals and substrates to support mitochondrial ATP production [6,59,70–73]. New evidence suggest that glycolytic and glycogenolytic enzymes, distributed intracellularly and associated with mitochondria, also have the ability to provide network capacity for transferring and distributing ATP produced in mitochondria [13,16]. Mitochondria, on the other side, can be interconnected providing cable properties for conduction of membrane potential along mitochondrial reticulum from precapillary area to inside muscle fibers as was elegantly demonstrated in Skulachev’s laboratory back in 70’s and 80’s [74]. However, ATP still needs to be exported from narrow mitochondrial cristae channels and delivered to cellular ATPases by facilitated diffusion or ligand conduction mechanisms [16]. Trans-mitochondrial cristae arrangement and phosphotransfer enzymes may facilitate navigation of ATP molecules out of mitochondrial cluster [16,75]. In this regard, deletion of intermembrane adenylate kinase AK2 isoform compromises ATP export and is embryonically lethal suggesting critical significance of phosphotransfer in facilitating ATP diffusion [76,77]. To this end, each heart muscle contraction, associated with ATPase activity, triggers precise and coordinated flux changes in coupled reaction systems maintaining almost constant metabolite levels [16,68]. Alteration of phosphotransfer fluxes, mostly in CK and AK systems, has been demonstrated under ischemic conditions and heart failure associated with poor contractile performance of the failing myocardium [7,9,20]. As was suggested previously [7,9], systemic accumulation of defects at various steps of the myocardial energetic system may compromise the ability to adequately restore electrical stability in the face of induced AF.

In summary, our data demonstrate that systemic alterations in ATP production and consumption and phosphotransfer-mediated energetic communication, and mitochondrial substrate supply processes underlie energetic limitation of the aging atrial myocardium. Aging induced decline in AK, CK, and glycolytic phosphotransfer circuits along with alpha-glycerophosphate shuttle, which are essential parts of myocardial bioenergetics infrastructure, hindering energetic communication and ATP cycling. Due to the tight relationship between myocardial energetic dynamics and cardiac electrical activity [21–24,27,65], these metabolic perturbations could increase vulnerability of aging atria to fibrillation, stroke, and sudden cardiac death. Potentiation of adrenergic signaling and associated Ca^2+^ cycling, such as occurs during physical activity, had beneficial effects on aging atrial bioenergetics system indicating potential of targeted prevention or slowing decline in specific energetic circuits to maintain quality of life.

## Supporting Information

S1 Table Mean values of phosphometabolite dynamics in adult and aging atrial myocardium. Data are expressed as % of oxygen replaced/min and represented as mean ± SEM (n = 6–8). Pi, inorganic phosphate; ATP γ-phosphoryl: phosphate at the gamma position of adenosine triphosphate; ADP β-phosphoryl, phosphate at the beta position of diphosphate; CrP, Creatine phosphate; G6P, glucose-6-phosphate; G1P, glucose-1-phosphate; G3P, glycerol-3-phosphate. Student’s t-Test was used to determine the significance between groups (p<0.05).
(DOCX)

S2 Table Mean values of first, second, third, and fourth ^18^O-atom incorporation into Pi signifying cycles of ATP/Pi exchange between ATP consumption and synthesis sites. Data are expressed as % of oxygen replaced/min and represented as mean ± SEM (n = 10–17). Student’s t-Test was used to determine the significance between groups (p<0.05).
(DOCX)

S3 Table Mean values of energy metabolite turnover rates and activity of corresponding metabolic pathways. Data are represented as mean ± SEM, n = 5–6. Phosphometabolites turnover times were calculated using the formula: *p*_*t*_(phosphometabolite) = (1-2^-N^)×p([^18^O]H_2_O), where *p*_*t*_(phosphometabolite) is a fraction of ^18^O-labeled phosphometabolite at given time *t*, N is equal to the number of turnover cycles observed during incubation period, and p([^18^O]H_2_O) is a fraction of ^18^O in media water as described in Materials and Methods. Abbreviations as in S1 Table. Student’s t-Test was used to determine the significance between groups (p<0.05).
(DOCX)
