# Supplementary material for: Decline of Phosphotransfer and Substrate Supply Metabolic Circuits Hinders ATP Cycling in Aging Myocardium
Source: PLoS One. 2015 Sep 17;10(9):e0136556. doi: 10.1371/journal.pone.0136556 (PMC4574965; doi:10.1371/journal.pone.0136556)
Supplement: S1 Table — Data are expressed as % of oxygen replaced/min and represented as mean ± SEM (n = 6–8). Pi, inorganic phosphate; ATP γ-phosphoryl: phosphate at the gamma position of adenosine triphosphate; ADP β-phosphoryl, phosphate at the beta position of diphosphate; CrP, Creatine phosphate; G6P, glucose-6-phosphate; G1P, glucose-1-phosphate; G3P, glycerol-3-phosphate. Student’s t-Test was used to determine the significance between groups (p<0.05). (DOCX) [file pone.0136556.s001.docx]

**S1 Table. Mean values of phosphometabolite dynamics in adult and aging atrial myocardium.** Data are expressed as % of oxygen replaced with ^18^O/min and represented as mean ± SEM (n=6-8). Pi, inorganic phosphate; ATP γ-phosphoryl: phosphate at the gamma position of adenosine triphosphate; ADP β-phosphoryl, phosphate at the beta position of diphosphate; CrP, Creatine phosphate; G6P, glucose-6-phosphate; G1P, glucose-1-phosphate; G3P, glycerol-3-phosphate. Student’s t-Test was used to determine the significance between groups (p<0.05).

| **Phosphometabolites** | **Adult**  **(6 month)** | **Adult (+ISO)**  **(6 month)** | **Aged**  **(24 month)** | **Aged (+ISO)**  **(24 month)** | **Group comparison (p-values)** | | |
| --- | --- | --- | --- | --- | --- | --- | --- |
| **(% of oxygen replaced/min)** | **Mean±SEM** | **Mean±SEM** | **Mean±SEM** | **Mean±SEM** | **Adult vs. Aged** | **Adult vs. Adult (+ISO)** | **Aged vs.**  **Aged (+ISO)** |
| Pi[^18^O] labeling rate | 22.672±1.642 | 25.320±1.546 | 12.608±1.239 | 16.780±0.996 | 0.001 | 0.267 | 0.025 |
| ATP[^18^O] γ-phosphoryl labeling rate | 38.765±3.803 | 48.333±5.389 | 28.984±3.289 | 32.220±1.833 | 0.099 | 0.177 | 0.455 |
| ADP[^18^O] β-phosphoryl labeling rate | 15.682±0.723 | 19.592±1.638 | 7.803±1.131 | 12.632±1.626 | 0.000 | 0.054 | 0.035 |
| CrP[^18^O] labeling rate | 60.200±0.641 | 59.248±6.652 | 39.910±2.708 | 48.370±3.023 | 0.001 | 0.917 | 0.033 |
| G6P[^18^O] labeling rate | 23.623±1.632 | 23.510±1.641 | 17.194±1.869 | 18.078±0.989 | 0.034 | 0.962 | 0.713 |
| G1P[^18^O] labeling rate | 2.872±0.905 | 4.018±0.852 | 3.176±1.400 | 4.146±1.478 | 0.872 | 0.401 | 0.675 |
| G3P[^18^O] labeling rate | 11.073±1.703 | 11.443±0.836 | 6.622±1.077 | 5.673±0.500 | 0.502 | 0.203 | 0.992 |
| Pi[^18^O]/γ-ATP[^18^O] labeling ratio | 0.600±0.047 | 0.484±0.037 | 0.408±0.046 | 0.528±0.049 | 0.030 | 0.100 | 0.167 |
