# Supplementary material for: Decline of Phosphotransfer and Substrate Supply Metabolic Circuits Hinders ATP Cycling in Aging Myocardium
Source: PLoS One. 2015 Sep 17;10(9):e0136556. doi: 10.1371/journal.pone.0136556 (PMC4574965; doi:10.1371/journal.pone.0136556)
Supplement: S2 Table — Data are expressed as % of oxygen replaced/min and represented as mean ± SEM (n = 10–17). Student’s t-Test was used to determine the significance between groups (p<0.05). (DOCX) [file pone.0136556.s002.docx]

**S2 Table. Mean values of first, second, third, and fourth ^18^O-atom incorporation into Pi signifying cycles of ATP/Pi exchange between ATP consumption and synthesis sites.** Data are expressed as % of oxygen replaced with ^18^O/min and represented as mean ± SEM (n=10-17). Student’s t-Test was used to determine the significance between groups (p<0.05).

| **ATP cycling** | **Adult**  **(6 month)** | **Age**  **(24 month)** | **Group comparison**  **(p-values)** |
| --- | --- | --- | --- |
| **1^st^ cycle (^18^O_1_)** | 13.743±0.584 | 11.076±0.847 | 0.035 |
| **2^nd^ cycle (^18^O_2_)** | 5.060±0.507 | 3.552±0.168 | 0.002 |
| **3^rd^ cycle (^18^O_3_)** | 0.645±0.121 | 0.292±0.032 | 0.002 |
| **4^th^ cycle (^18^O_4_)** | 0.064±0.010 | 0.049±0.004 | 0.140 |
