# Supplementary material for: Decline of Phosphotransfer and Substrate Supply Metabolic Circuits Hinders ATP Cycling in Aging Myocardium
Source: PLoS One. 2015 Sep 17;10(9):e0136556. doi: 10.1371/journal.pone.0136556 (PMC4574965; doi:10.1371/journal.pone.0136556)
Supplement: S3 Table — Data are represented as mean ± SEM, n = 5–6. Phosphometabolites turnover times were calculated using the formula: p t(phosphometabolite) = (1-2-N)×p([18O]H2O), where p t(phosphometabolite) is a fraction of 18O-labeled phosphometabolite at given time t, N is equal to the number of turnover cycles observed during incubation period, and p([18O]H2O) is a fraction of 18O in media water as described in Materials and Methods. Abbreviations as in S1 Table. Student’s t-Test was used to determine the significance between groups (p<0.05). (DOCX) [file pone.0136556.s003.docx]

**S3 Table. Mean values of energy metabolite turnover rates and relative activities of corresponding metabolic pathways.** Data are represented as mean ± SEM (n=5-6). Phosphometabolites turnover times were calculated using the formula: *p_t_*(phosphometabolite)=(1-2^-^***^N^***)×p([^18^O]H_2_O), where *p_t_*(phosphometabolite) is a fraction of ^18^O-labeled phosphometabolite at given time *t*, N is equal to the number of turnover cycles observed during incubation period, and p([^18^O]H_2_O) is a fraction of ^18^O in media water as described in Materials and Methods. Abbreviations as in S1 Table. Student’s t-Test was used to determine the significance between groups (p<0.05).

| **Metabolites/Pathways** | **Adult**  **(6 month)** | **Adult (+ISO)**  **(6 month)** | **Aged**  **(24 month)** | **Aged (+ISO)**  **(24 month)** | **Group comparison (p-values)** | |
| --- | --- | --- | --- | --- | --- | --- |
|  | **Mean±SEM** | **Mean±SEM** | **Mean±SEM** | **Mean±SEM** | **Adult vs. Aged** | **Adult (+ISO) vs. Aged (+ISO)** |
| **G3P[^18^O]/Substrate shuttle** | 0.241±0.043 | 0.301±0.036 | 0.135±0.032 | 0.134±0.015 | 0.053 | 0.000 |
| **G1P[^18^O]/Glycogenolysis** | 0.088±0.021 | 0.101±0.022 | 0.100±0.036 | 0.106±0.039 | 0.814 | 0.916 |
| **β ADP[^18^O]/Adenylate kinase** | 0.367±0.019 | 0.478±0.048 | 0.168±0.027 | 0.290±0.041 | 0.000 | 0.014 |
| **β ATP[^18^O]/Adenylate kinase** | 0.395±0.054 | 0.509±0.106 | 0.166±0.043 | 0.355±0.091 | 0.012 | 0.323 |
| **G6P[^18^O]/Glycolysis** | 0.472±0.038 | 0.470±0.039 | 0.330±0.040 | 0.346±0.022 | 0.037 | 0.030 |
| **γ ATP[^18^O]/ATP synthesis** | 0.722±0.094 | 0.996±0.161 | 0.502±0.063 | 0.564±0.039 | 0.104 | 0.042 |
| **Pi[^18^O]/ATPases** | 0.493±0.047 | 0.634±0.028 | 0.268±0.029 | 0.366±0.024 | 0.002 | 0.000 |
| **CrP[^18^O]/Creatine kinase** | 1.260±0.056 | 1.285±0.182 | 0.671±0.082 | 0.935±0.091 | 0.000 | 0.076 |
